# Systems analysis reveals neuregulin-1 control of cardiomyocyte size and shape mediated by distinct PI3K and p38 pathways

**DOI:** 10.1101/2025.10.01.679873

**Published:** 2025-10-03

**Authors:** Pichayathida Luanpaisanon, Philip M. Tan, Karen A. Ryall, James O’Hearn, Laura A. Woo, Bryana N. Harris, Bethany Wissmann, Alexander Paap, Matthew Rhoads, Jeffrey J. Saucerman

**Affiliations:** 1 Department of Biomedical Engineering, University of Virginia, Charlottesville VA, 22908; 2 Division of Cardiovascular Medicine, University of Virginia, Charlottesville VA, 22908

## Abstract

Cardiac hypertrophy is a common precursor to heart failure, but its cellular manifestations—changes in cardiomyocyte size and shape—are regulated by poorly understood signaling networks. Here, we combined high-content morphological profiling, proteomics, and systems modeling to characterize the diverse forms of hypertrophy induced by angiotensin II, endothelin-1, insulin growth factor-1, and neuregulin-1 (Nrg1). Reverse-phase protein array profiling and partial least squares regression modeling revealed that AKT, GSK3, and MAPK signaling are differentially regulated by hypertrophic agonists and are predictive of distinct phenotypic outcomes. Nrg1 uniquely induced cardiomyocyte elongation in both neonatal rat and human iPSC-derived cardiomyocytes, in addition to increasing cell area. Pharmacological perturbations demonstrated that Nrg1-induced elongation and area expansion both require PI3K activity, whereas p38 selectively mediates cell area. A logic-based network model incorporating dual-specificity phosphatases were sufficient to capture the amplifying PI3K and transient p38 signaling dynamics driving phenotypic changes. Together, these results identify distinct signaling cascades by which Nrg1 coordinates cardiomyocyte size and shape, providing mechanistic insight into how hypertrophic remodeling can be differentially regulated. This systems approach provides new insight into the pathways that drive distinct forms of cardiomyocyte hypertrophy, highlighting opportunities to selectively target maladaptive remodeling in heart failure.

## Introduction

Cardiac hypertrophy is a compensatory response of the heart to increased workload, characterized by an increase in cardiomyocyte size, thickening of ventricular walls, and increased cardiac mass. Cardiomyocytes (CMs) account for approximately 30% of total cell number but contribute to 70–80% of the heart’s mass [1], [2]. During the compensatory stage of hypertrophy, cardiomyocytes undergo considerable changes in cell size and shape initiated by complex signaling cascades [2]. While cardiomyocyte cross-sectional cell area has been widely accepted as a proxy for hypertrophic growth, the mechanisms governing changes in CM shape remain poorly understood. For example, pathologic growth of the myocardium can induce concentric remodeling that results in cardiomyocyte growth in a cross-sectional area [3], [4]. Alternatively, some pathologic stimuli can elicit an eccentric hypertrophy through a predominate lengthening of individual CMs, which can be a key factor in the transition to heart failure [5], [6]. At the same time, physiological cues such as resistance and endurance exercise induce concentric or eccentric hypertrophy, respectively, in which the molecular pathways driving these distinct forms of growth are poorly understood [7], [8].

CMs respond to both mechanical and chemical loadings, such as growth cues, including cytokines, growth factors, catecholamines, vasoactive peptides, and hormones. Among the most well-characterized growth factors to regulate hypertrophy are angiotensin II (AngII), endothelin-1 (ET1), neuregulin-1 (Nrg1), and insulin growth factor-1 (IGF1) [9], [10], [11], [12], [13]. Some studies indicate that CM size may be regulated by shared signaling pathways, whereas CM shape and sarcomeric organization are regulated by distinct pathways [14]. Previous study from our lab used a high-content microscopy screen to identify specific growth factors associated with CM shapes and gene expression signatures. Particularly, Nrg1 induced both CITED4 mRNA abundance and CM elongation, with CITED4 knockdown further accentuating elongation [13]. However, in most cases it is still unclear how interconnected signaling pathways induce distinct cell features associated with hypertrophy, especially for cell shape.

To identify pathways that distinctly regulate cardiomyocyte size, shape, and gene expression, we performed a proteomic screen under AngII, ET1, Nrg1, and IGF1 conditions where we previously found diverse cardiomyocyte phenotypes. Proteomic analysis revealed distinct cell states depending on treatment and timepoint. We further mapped the proteomic data to the previous phenotypic screen to infer signaling modules and pathways. Finally, we validated the predicted mediators of Nrg1-dependent cardiomyocyte size and shape using rodent and human iPSC-derived cardiomyocytes.

## Results

### Neuregulin-1 induces elongation of rat and human cardiomyocytes

**Figure 1.**
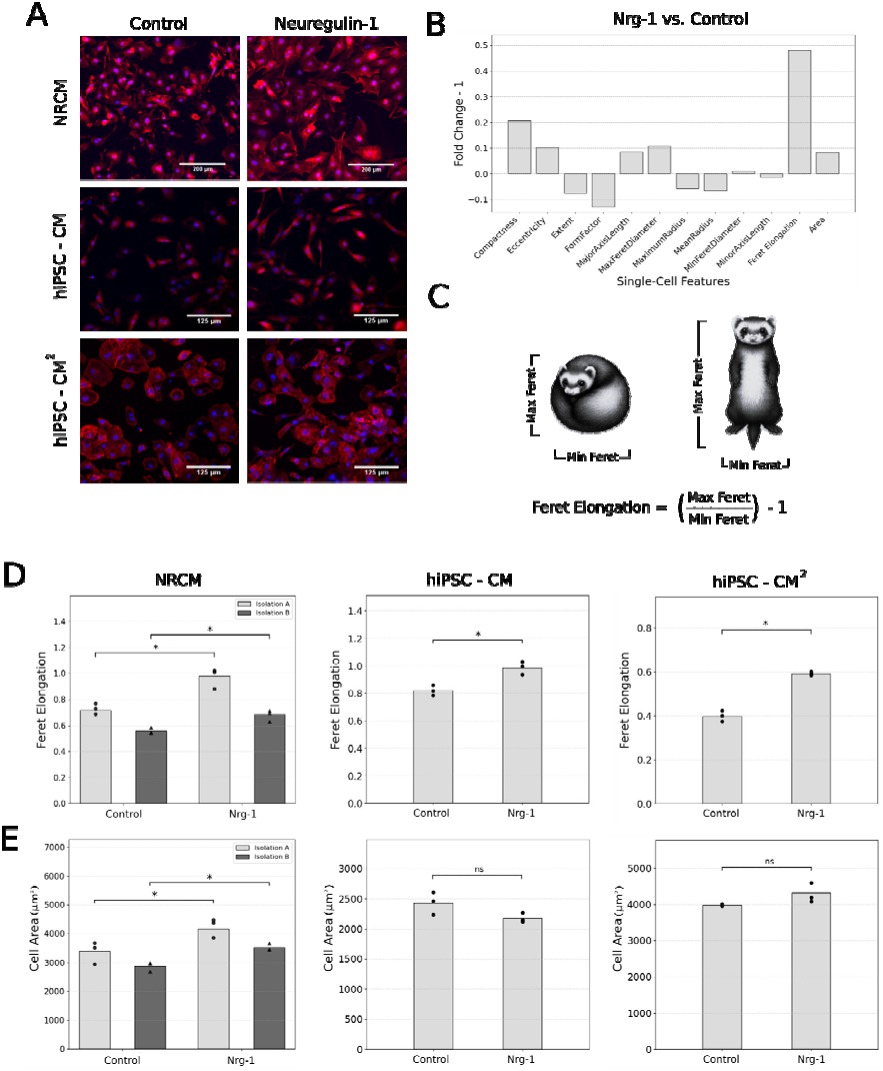
Neuregulin induces elongation of rat and human-derived cardiomyocytes. (A) Representative images of neonatal rat cardiomyocytes (NRCM), hiPSC iCell cardiomyocytes, and hiPSC iCell Cardiomyocytes^2^ at 48hr after treatment with control or 100ng/mL Neuregulin (Nrg1). Cardiomyocyte marker (α-actinin) and nuclei (DAPI) are shown. (B) Profiling 12 single-cell morphological features for sensitivity to Nrg1-treatment compared to control NRCMs, quantified as fold-change from control minus 1. (C) Schematic illustrating the Feret Elongation metric. (D) Change in Feret Elongation in response to Nrg treatment in NRCM, hiPSC-CM, and hiPSC-CM^2^. (E) Change in cell area in response to Nrg treatment in NRCMs, hiPSC-CM, and hiPSC-CM^2^. Each point (dot or triangle) reflects the mean of all cells per image, and the bar reflects mean across images. Asterisks denote p < 0.05 from two-way ANOVA followed by Tukey post-hoc tests in NRCMs and Student’s t-tests in hiPSC-CMs. “ns” denotes not significant.

In a previous screen of hypertrophic ligands, we found that Nrg1 was unique in its strong induction of cardiomyocyte elongation while also inducing biochemical responses consistent with physiological growth, such as CITED4 [13]. Those studies were performed with neonatal rat cardiomyocytes in a live-cell assay, where we tracked the subpopulation (~5% of cells) that transiently expressed GFP under a cardiac-specific cTnT promoter. While sensitive to single-cell changes, a limitation was that this subpopulation may not be representative. To determine whether Nrg1 also induced size and shape changes in a broader population of cells, we treated rat neonatal cardiomyocytes and two commercial preparations of human iPSC-derived cardiomyocytes (iCell hiPSC-CMs and hiPSC-CM^2^ from FujiFilm) (Figure 1A). At 48 h, we fixed and immune-stained the cells for α-actinin, followed by high-content cell segmentation and extraction of cell morphological features. By high-content analysis, we profiled 12 single-cell size and shape features for sensitivity to Nrg1 response. Of these, the most sensitive response to Nrg1 was by a new metric we term Feret Elongation (Figure 1B). Feret Elongation is more sensitive to changes in the overall extent of length and width of cardiomyocytes (Figure 1C and Supplementary Figure 1). In contrast, other shape measures we and others have used previously compare cells to circles (form factor) or ellipses (eccentricity). Comparison of cell types demonstrated consistent Nrg1-dependent elongation of NRCMs, hiPSC-CMs and hiPSC-CM^2^ (Figure 1D). Nrg1 also increased cell area in NRCMs but not hiPSC-CMs and hiPSC-CM^2^ (Figure 1E).

### Proteomic analysis exposes unique time-dependent responses to specific growth factors

**Figure 2.**
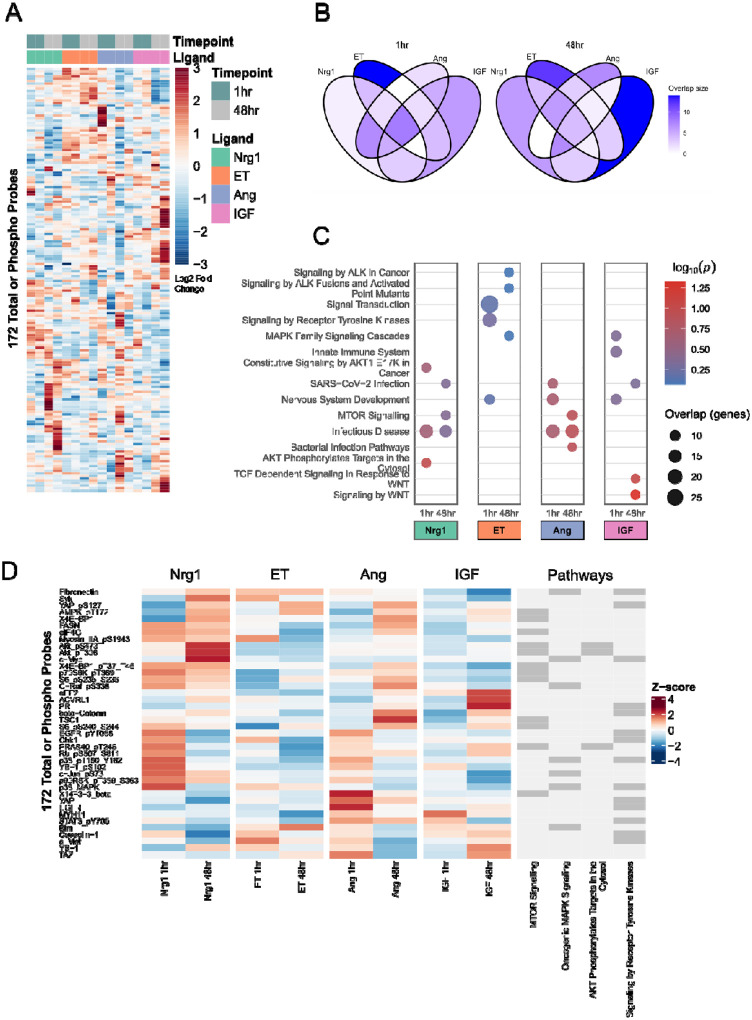
Hypertrophic ligands induce diverse changes in protein abundance and phosphorylation. (A) Hierarchical clustering of RPPA data from 172 total/phospho probes following 1 hr and 48 hr treatment with Nrg1 (10 ng/mL), Ang II (1 μM), ET-1 (100 nM), or IGF-1 (10 nM) (log2 fold-change compared to control). (B) Venn diagrams of top 20% most responsive proteins under each treatment at 1 hr vs 48 hr. (C) Enrichr Reactome pathway enrichment analysis, showing the top 3 enriched pathways with overlap genes ≥ 10. (D) Differentially expressed or phosphorylated proteins corresponding to with the most enriched Reactome pathways for each treatment.

Because pathways that regulate cardiomyocyte elongation are not well characterized, we systematically profiled protein abundance and phosphorylation under treatments that influence cardiomyocyte morphology. Neonatal rat ventricular cardiomyocytes (NRCMs) were treated with Nrg1 (10 ng/mL), AngII (1 μM), ET (100 nM), or IGF (10 nM) and sampled at 1 hr and 48 hr. Reverse-phase protein array (RPPA) analysis quantified total and phospho-protein levels across 172 antibody probes. Heatmaps summarizing this data revealed both time- and ligand-specific proteomic responses compared to serum-free controls (Figure 2A). To highlight dynamic changes that are unique among treatment groups, from each group we selected the top 20% proteins most expressed at 1 hr and 48 hr.

Venn diagrams revealed that many responses were largely shared across ligands at 1 hr, but proteomic responses became progressively more ligand-specific by 48 hr (Figure 2B). For example, Nrg1 and AngII shared 22 differentially-expressed proteins at 1 hr and none at 48 hr. ET was the only condition with a substantial set of unique proteins already at 1 hr. Pathway enrichment using Enrichr (Reactome database) [16, 17] identified enrichment of differentially expressed proteins associated with AKT/mTOR signaling under Nrg1 treatment, receptor-tyrosine-kinase–related pathways and MAPK signaling for ET treatment, infection/immune-related pathways for AngII treatment, and developmental signaling and WNT signaling for IGF treatment (Figure 2C). Exploration of differentially expressed proteins associated within each of these enriched pathways revealed that Nrg treatment induces early activation of the Ras/MAPK signaling—elevated phosphorylation of ERK/MAPK, p38, JNK, c-Jun, and c-Raf—at 1 hr, whereas AKT was significantly upregulated by 48 hr (Figure 2D).

### Data-driven model identifies relationships between the hypertrophic ligands with changes in protein expression, gene expression, and cardiomyocyte size and shape

The proteomic experiments identified distinct signatures of protein abundance and phosphorylation, but these measurements alone are insufficient to determine which proteins most closely associate with cardiomyocyte morphology and gene expression. To address this gap, we mapped proteomic responses to cardiomyocyte morphology and transcriptomic changes obtained from our prior phenotypic screen [13] using a partial least squares regression (PLSR) model. PLSR provides a powerful framework for dimensionality reduction and visualization of correlations, thereby offering insight into the organization of signaling networks [15].

**Figure 3.**
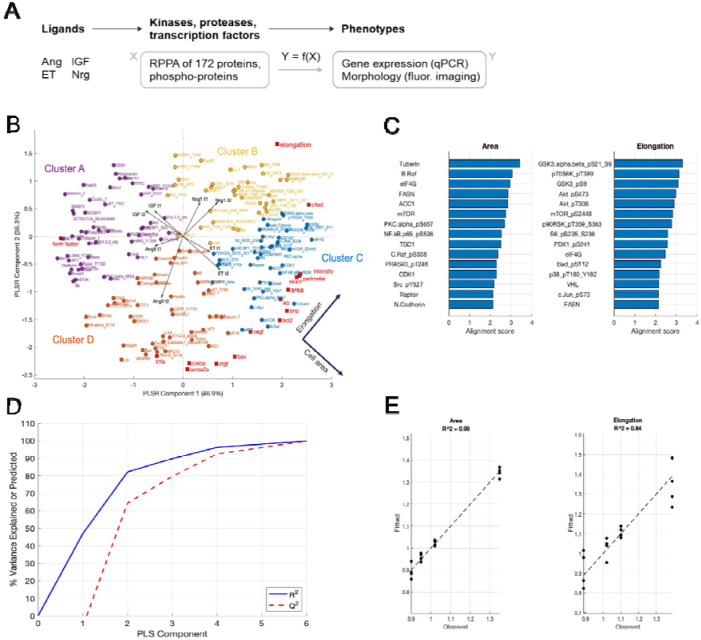
Partial least squares regression (PLSR) model predicts unique signaling pathways associated with CM size and elongation. (A) Overview of experimental design for PLSR model, in which the goal is to identify predictive relationships from protein and phospho-protein expression (predictor block) to gene expression and morphological changes (response block). Protein levels were measured by RPPA 48 hours after treatment, gene expression was measured by qPCR 48 hours after treatment, and morphology of individual cells was tracked by fluorescent microscopy from before treatment to 48 hours after treatment. (B) PLSR loadings are plotted for protein expression (predictor block; circle markers) and for gene expression and morphology (response block; square markers). Unique signaling pathways are grouped together via K-means cluster analysis. Middle arrows show orientation and relative magnitude of PLSR scores for ligand inputs at two timepoints (1hr and 48hr after treatment). (C) Alignment scores of cell area and elongation in PLS latent space shown in panel B. (D) PLSR model performance, including percent variance explained (R^2^) and leave-one-out cross-validation to estimate predictive value (Q^2^). (E) Observed-versus-fitted plots for area and elongation.

This PLSR model was created by designating the RPPA data as the predictor block (172 probes across four hypertrophic ligands 1 hr and 48 hr) and the phenotypic outcomes as the response block (11 mRNAs and four morphology metrics [13]) (Figure 3A). All outputs were centered and scaled by z-score across ligands. The first two latent variables accounted for 82% of the total variance, enabling visualization of RPPA and phenotypic loadings together with ligand scores in a shared latent space (Figure 3B). Consistent with trends observed in the RPPA dataset alone, each treatment induced distinct contributions in the latent space, reflecting ligand-specific signaling programs (Figure 3B).

Of note, Nrg1 aligned strongly with cardiomyocyte elongation and CITED4 expression, consistent with our prior findings [13]. Further, unsupervised k-means clustering of protein loadings revealed a distinct module of protein expression and phosphorylation (Cluster B) that are associated with cardiomyocyte elongation. Indeed, Cluster B is composed of many components of the PI3K pathway including phospho-PI3K, phospho-Akt, phospho-PDK1, phospho-GSK3, phospho-mTOR, and phospho-p70S6K. Also, within Cluster B and correlated with elongation are several MAPK signaling proteins such as phospho-p38, phospho-MEK1, phospho-MAPK, and phospho-p90RSK (Figure 3B).

To further dissect proteins associated with cell area vs. elongation, we computed alignment scores. While cell area was most aligned with abundance of proteins such as tuberin and bRaf, elongation was aligned with phosphorylation of proteins close to PI3K/Akt signaling such as GSK3α, p70S6K, Akt, mTor, and p38 (Figure 3C). To assess PLSR model performance, we assessed two complementary metrics: (i) percent variance explained (R^2^) and (ii) leave-one-out cross-validation to estimate predictive value (Q^2^) (Figure 3D). Observed-versus-fitted analyses for individual phenotypes demonstrated high predictive accuracy, with R^2^ values of 0.99 for cell area and 0.84 for elongation (Figure 3E). Taken together, these findings demonstrate that Nrg1-induced changes in phenotype are most strongly associated with PI3K/Akt and MAPK signaling modules, highlighting these pathways as key regulators of cardiomyocyte elongation.

### PI3K mediates Nrg1-induced elongation and cell area, while p38 mediates Nrg1-induced increase in cell area

Proteomic measurements and PLSR modeling indicated that signatures of protein phosphorylation within the PI3K/AKT and MAPK pathways were closely aligned with cell elongation in response to Nrg1 treatment. This prompted us to hypothesize that Nrg1-induced hypertrophic remodeling is mediated by PI3K and p38 signaling. PI3K was selected given its role as a central upstream regulator of mTOR and AKT signaling, while p38 was chosen as the top differentially expressed and most highly ranked MAPK protein.

**Figure 4.**
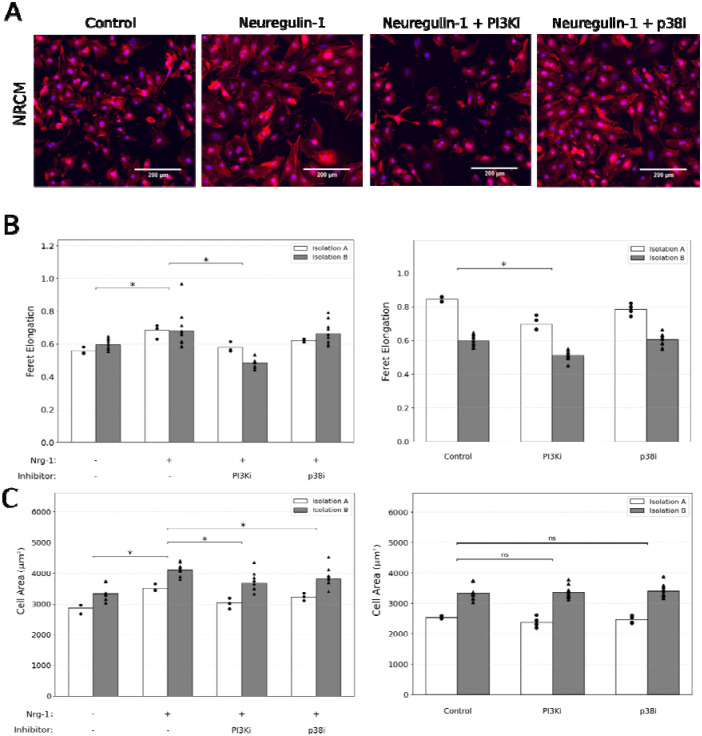
PI3K and p38 distinctly mediate Neuregulin-induced changes in cardiomyocyte morphology. (A) Representative images of neonatal rat cardiomyocytes (NRCMs) under four conditions: Control, 100ng/mL Neuregulin-1 (Nrg1), Nrg1 + 10uM LY294002 (PI3Ki), and Nrg + 10uM SB203580 (p38i). Cardiomyocyte marker (α-actinin) and nuclei (DAPI) are shown. (B) Quantification of Feret elongation and cell area (C) across two independent biological isolations. Left panels: Nrg1 treatment increases elongation and cell area relative to Control. Co-treatment with PI3Ki reduces both effects, whereas p38i reduces the increase in cell area but not elongation. Right panels: PI3Ki and p38i were tested separately in the absence of Nrg to assess their standalone effects. Data is shown as mean across images. Each point (dot or triangle) represents the average measurement per image. Asterisks denote p < 0.05 from two-way ANOVA followed by Tukey post-hoc tests in NRCMs and Student’s t-tests in hiPSC-CMs. “ns” denotes not significant.

To test this hypothesis, we treated NRCMs with 10 ng/mL Nrg1 and applied pharmacological inhibitors for PI3K (10 μM LY294002) or p38 (10 μM SB203580). Consistent with our prior NRCM experiments, Nrg1 treatment increased both cardiomyocyte elongation and cell size (Figure 4A–C). PI3K inhibition significantly attenuated Nrg1-induced elongation and reduced overall cell area, indicating a broad role in Nrg1-mediated hypertrophy (Figure 4A–C). In contrast, p38 inhibition reduced the Nrg1-dependent increase in cell area without significantly affecting elongation (Figure 4A–C).

### Network modeling predicts signaling cascades sufficient to regulate elongation and cell area following Nrg1 simulation

The above experimental studies revealed that Nrg1 induces protein changes associated with cardiomyocyte elongation and that elongation and cell area, and that these phenotypes differentially require PI3K and p38 activity. We next asked whether our experimentally measured dynamics of PI3K and p38 are sufficient to predict the distinct network dynamics of elongation and cell area, with and without inhibitors of those pathways.

To address this question, we developed a logic-based computational model to formalize our network hypothesis, integrating data from RPPA measurements, phenotypic assays, and PLSR predictions. In this network model, Nrg1 was assumed to act through ERBB receptors, activating both PI3K/Akt/elongation/area and Ras/p38/area cascades. P38 activity was further constrained by negative regulation through Dual-specificity phosphatase (DUSP), which we hypothesized would be sufficient to predict the adaptive decline in p38 phosphorylation observed experimentally. We implemented this network structure in Netflux, a logic-based differential equation modeling platform [16].

**Figure 5.**
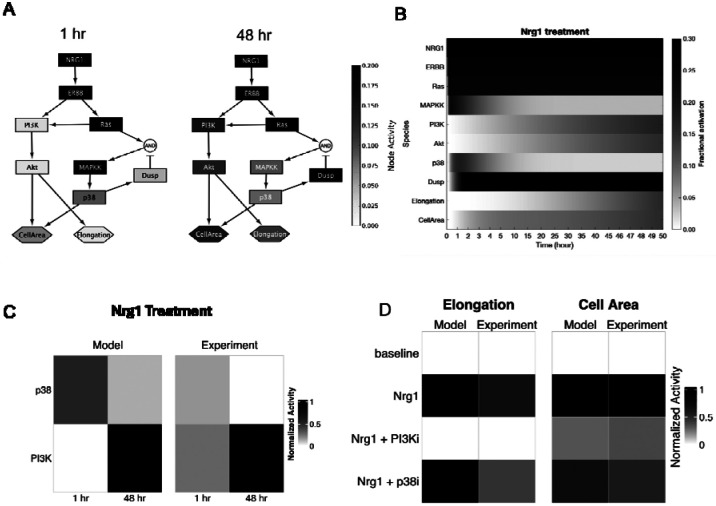
Network model identifies mechanisms sufficient for distinct regulation of cell size and shape. (A) Logic-based network model of Nrg1 signaling, with nodes colored based on their normalized activity at 1 hr and 48 hr following Nrg1 treatment. (B) Simulated response of the network in response to Nrg1. (C) Comparison of phosphorylation levels between model predictions and experiments (Akt phosphorylation: average of pS473 and pT308; p38 phosphorylation: pT180/Y182), normalized by min/max values per experiment (D) Comparison of cell area and elongation between model predictions and experimental results, normalized by min/max values per experiment.

Simulations of Nrg1 treatment predicted network-wide dynamic responses, including early activation of ERBB/Ras/p38 signaling, followed by a subsequent rise in PI3K signaling and DUSP-induced decline in p38 signaling (Figure 5A–B). As seen in the RPPA experiments, the model predicted that Akt phosphorylation rose slowly, while p38 phosphorylation was transient (Figure 5C). Perturbation simulations further validated the model. As measured experimentally, PI3K inhibition attenuated Nrg1-induced elongation and decreased cell area, while p38 inhibition selectively reduced cell area response without strongly impacting elongation (Figure 5D). Together, these findings demonstrate that simplified network model grounded in our experimental data recapitulates the differential pathway contributions of PI3K and p38 to both cell elongation and area.

## Discussion

A quantitative understanding of cardiomyocyte size and shape regulation is critical for elucidating the molecular pathways that govern cardiac hypertrophy. A wide range of studies have focused on molecular regulators of cardiomyocyte size, such the calcineurin/NFAT pathway [17]. In contrast, pathways that distinctly control cardiomyocyte shape, such cardiomyocyte elongation often seen after volume overload or endurance exercise, remain underexplored. Here, we show that Nrg1 regulates both size and shape across neonatal rat cardiomyocytes and two lines of hiPSC-derived cardiomyocytes. Using a new metric of cell elongation termed Feret elongation, we identified that in addition to increasing cell area, Nrg1 is a potent inducer of cardiomyocyte elongation.

To identify pathways associated with cardiomyocyte size and shape, we performed proteomic screens in response to hypertrophic ligands ET1, AngII, Nrg1, and IGF1. Partial Least Squares Regression modeling revealed patterns of protein expression and phosphorylation that are predictive of gene regulation and cell morphology. These predictions guided subsequent perturbation experiments, which identified that Nrg1 mediated PI3K-dependent cell elongation while both PI3K and p38 contribute to cell area. Integrating these data into a logic-based network model demonstrated sufficient mechanisms by which sustained PI3K drives cell elongation, while negative feedback by dual-specificity phosphatases (DUSPs) [18], [19] attenuates p38-driven regulation of cell area. Together, these results highlight how distinct signaling cascades differentially regulate cardiomyocyte size versus shape.

Our proteomic-phenotypic analysis aligns with prior studies. For example, we found that ET1 induced a distinct cluster of protein responses associated with receptor tyrosine kinase and mitogen-activated protein kinase signaling pathways (Figure 2D) that were predictive of pathological markers CTGF and Bax mRNAs as well as increased cell area [13]. In contrast to cell area, only a few pathways have been shown to drive cardiomyocyte elongation (e.g. CT1/LIF, MEK5, endurance exercise) or protect against it (CITED4, ERK1/2) [11], [20], [21], [22], [4], [23], [24], [25], [26]. Here by immunofluorescence and previously by live-cell imaging [13], we find that Nrg1 most substantially increased elongation. Notably, in neurons, Nrg1 is well characterized to promote neurite outgrowth and axonal elongation [27], underscoring its broader role regulating cell shape. Together, these findings decouple “size” from “shape” in cardiomyocytes: PI3K drives both, whereas p38 selectively regulates area.

Although Nrg1-mediated elongation was consistently observed across neonatal rat cardiomyocytes, hiPSC-CMs, and hiPSC-CM^2^ cells, *in vivo* studies are required to determine the extent to which these cell phenotypes and proteomic signatures drive physiological vs. pathological eccentric hypertrophy. Still, our data demonstrates that Nrg1 induces several responses associated with physiological hypertrophy including PI3K/Akt activation [7], CITED4 expression [28], [13], and enhanced sarcomere organization associated with increased maturity (Supplemental Figure 2) [7]. Further, we found Nrg1 downregulates pathological markers C/EBPβ, CTGF, and Bax [13]. Indeed, a number of studies in rodents and humans have shown that Nrg1 mediates beneficial aspects of endurance exercise, which induce a physiological eccentric hypertrophy [24], [29], [30], [31].

Nrg1 has previously been implicated in cardiac development and disease [32], [33]. In zebrafish, Nrg1 overexpression enhances cardiomyocyte proliferation and supports trabeculation—an essential morphogenetic process required for myocardial growth prior to coronary vessel formation. Disruption of trabeculation results in embryonic lethality or adult-onset dilated cardiomyopathy [34], [35]. In mammals, Nrg1-induced hypertrophy also may be accompanied by some degree of cardiomyocyte proliferation [35], [36], [37], [37], [38], [39]. In humans, chronic exercise has been associated with elevated Nrg1 and physiological hypertrophy [31]. Circulating Nrg1 also rises with heart-failure severity, consistent with a compensatory response to stress [40]. Recombinant human Nrg1 (Neucardin) has completed phase III trials as a treatment for chronic heart failure [41], although those results are not yet available. Phase II trials showed structural and functional improvements [42]. However, not all findings are favorable, with some evidence that Nrg1-induced hypertrophy and ejection fraction may be accompanied by reduced cardiac output [12]. Together, these observations underscore a context- and time-dependent role for Nrg1: acutely adaptive, but potentially maladaptive with chronic exposure.

These findings of distinct regulation of size and shape signaling are currently limited to an *in vitro* context, which motivates future studies. For example, do other more pathologically-associated stimuli of myocyte elongation (LIF, MEK5 [20], [43]) induce distinct pathway mechanisms from Nrg1, which appears more physiological? While we tested neonatal cardiomyocytes and two human iPSC-cardiomyocyte lines, it is of interest to expand this study to adult cardiomyocytes and subsequently to in vivo animal models. Future studies should test Nrg1 and downstream pathways implicated here in vivo, incorporating longitudinal analyses to distinguish adaptive from maladaptive remodeling. Examination of Nrg1-induced cell proteomic-phenotype signatures and mechanisms should be tested in endurance exercise [29], which involves physiological eccentric hypertrophy and Nrg1 secretion but incompletely characterized mechanisms. Integration of single-cell transcriptomics or spatial proteomics with machine learning image analysis may help identify how cell size and shape are coordinated at the tissue level.

In summary, this study identifies Nrg1 as a potent regulator of both cardiomyocyte elongation and area, mediated through distinct signaling pathways. PI3K signaling contributes to both elongation and cell area expansion, whereas p38 selectively drives cell area growth. These findings reveal that cardiomyocyte size and shape can be uncoupled mechanistically, advancing our understanding of how hypertrophic remodeling is orchestrated. Leveraging systems-level approaches to selectively modulate maladaptive elongation while preserving adaptive growth may open new therapeutic opportunities for the treatment of cardiac hypertrophy.

## Methods:

### Cell Culture

Neonatal rat cardiomyocytes were isolated from 1 to 2 day old Sprague Dawley rats using the Neomyts isolation kit (Cellutron, Baltimore, MD). Myocytes were cultured on 96-well Corning CellBIND plates (30,000 cells/well) coated with SureCoat (a combination of collagen and laminin, Cellutron) and in plating media (Dulbecco’s modified Eagle media, 17% M199, 10% horse serum, 5% fetal bovine serum, 100 U/mL penicillin, and 50 mg/mL streptomycin).

Human iPSC-derived cardiomyocytes (iCell hiPSC-CMs and hiPSC-CM^2^ from FujiFilm) were cultered on 96-well Corning CellBIND plates (10,000 cells/well) coated with SureCoat and in iCell plating media (FujiFilm). After 48 h of incubation with daily media change (iCell Maintenance Media, FujiFilm), the medium was replaced with Williams E Medium (Life Technologies, A1217601) supplemented with cocktail B supplement (Life Technologies, CM4000) serum-starved for at least 4 h before treatment.

All procedures were performed in accordance with the Guide for the Care and Use of Laboratory Animals published by the US National Institutes of Health and approved by the University of Virginia Institutional Animal Care and Use Committee.

### Immunofluorescent and high-content imaging

To prepare for immunofluorescent imaging, cardiomyocytes were first fixed with 4% paraformaldehyde for 20 min and then permeabilized with 0.1% Triton-X for 15 min. Cardiomyocytes were blocked with 1% bovine serum albumin in PBS for 1 hr, then treated with mouse anti-α-actinin primary antibody (Sigma-Aldrich Cat#A7811, RRID:AB_476766) at a concentration of 1:200 overnight. Cardiomyocytes were blocked with 5% goat serum in PBS for 1 hr, then Alexa Fluor-568-conjugated goat anti-mouse secondary antibody (Thermo Fisher Scientific Cat#A11031, RRID:AB_144696) at a concentration of 1:200 was applied for 1 hr. The cells were stained with DAPI prior to imaging.

High-content imaging was performed on the stained cardiomyocytes using an Operetta CLS High Content Analysis System. These images were processed using CellProfiler using a cellular segmentation algorithm developed previously and validated to within 5% of two independent manual segmentations [44], [45]. Median cell area was used as a representative measure of the cell population in each well, and cells with undetectable cytoplasm were not counted.

### Single-cell morphology features

To quantify cardiomyocyte shape features, single-cell morphological analysis was performed on CellProfiler-segmented images. Myocytes were filtered based on integrated intensity and percent of neighboring cells touching to ensure inclusion of only healthy, well-isolated cells for accurate shape quantification.

For each retained cell, 15 morphological metrics were measured, including 10 size metrics and 5 shape metrics. From two size metrics, a new shape metric was derived:

$$\text{Feret elongation}=\left(\text{Max Feret Diameter}/\text{Min Feret Diameter}\right)-1.$$

This metric (Feret Elongation) captures the degree of myocyte elongation at all angles, where a value of 0 corresponds to a perfectly non-elongated cell, and higher values indicate greater elongation. Feret elongation is well suited to quantify myocyte elongation because it is scale-independent, rotation-independent, and sensitive to differences between maximum length and minimum width. Scale independence means that the metric remains unchanged if a cell increases or decreases in size proportionally. Additionally, subtracting 1 from the Feret ratio normalizes cells to a baseline minimum width of 1, allowing for comparisons independent of size.

### RPPA proteomics analysis

Reverse Phase Protein Array (RPPA) analysis was performed to measure the phosphorylation state and changes in protein levels of 172 different proteins. RPPA is a high-throughput antibody-based method for measuring protein concentration similar to Western blots.

Neonatal rat ventricular myocytes were cultured in 24-well plates (500,000 myocytes/well). Four days after isolation, myocytes were rinsed and cultured in serum-free media containing 10 ng/mL Nrg1, 10 nM ET1, 1 μM Ang II, 10 nM IGF1, or serum (10% horse serum and 5% fetal bovine serum). Myocyte protein was isolated at two time points: 1 hour and 48 hours following administration of the ligands according to the protocol on the MD Anderson Cancer Center RPPA Core Facility website. Protein concentration was quantified using the Pierce 660 nM Protein Assay Kit (Thermo Scientific). Cell lysates were submitted to the MD Anderson Cancer Center for analysis. Data was collected from 2 independent myocyte isolations per condition, and all data points were normalized relative to serum-free condition for protein loading.

### Partial Least Square Regression (PLSR) model analysis

Partial Least Squares Regression (PLSR) analysis was performed using MATLAB to infer relationships between RPPA protein expression data and phenotypic screen outputs, which included gene expression and morphological features. The predictor block consisted of 172 proteins across 4 ligands, and the response block comprised 15 phenotypic outputs (11 mRNA transcripts and 4 morphology metrics) across the same ligands. Prior to constructing the model, both RPPA and phenotype data were log-transformed and standardized by computing z-scores across ligands to ensure comparability and eliminate scale differences. PLSR was implemented using MATLAB’s plsregress function. To determine the optimal number of latent components, models with up to six components were tested. The model performance was evaluated using both the cumulative coefficient of determination (**R**^**2**^) and predictive ability (**Q**^**2**^). These metrics were calculated across components and visualized in a variance-explained plot, allowing identification of the number of components that maximized model performance while minimizing overfitting.

All MATLAB scripts used for data preprocessing, model fitting, cross-validation, and visualization are available on GitHub at https://github/saucermanlab/neuregulin.

### Network model construction

A knowledge-based computational model of the distinct cardiomyocyte size and shape signaling network was developed from known direct molecular interaction from the literature, RPPA measurements, phenotypic assays, and PLSR predictions. We employed the logic-based differential equation (LDE) approach [46] to build a predictive framework to explore cardiomyocyte cell area and elongation regulation. A normalized Hill function modeled activation of each node by its upstream reactions. We modeled pathways crosstalk by continuous gates representing “OR” and “AND” logic [46]. The OR gates were used for reactions that modify the node regardless of others and the AND gates for reactions affecting each other. The default reaction parameters in the model are reaction weight (W = 1), half-maximal effective concentration (EC_50_ = 0.5), and Hill coefficient (n = 1.223). The time constant (τ =0.1), initial activation (Yinit = 0), and maximal activation (Ymax = 1) regulate the dynamics of signaling nodes. In conditions with Nrg1 treatment, its input reaction weight was increased (0.02 → 0.3). Further, time constants (tau parameters) were manually calibrated to fit the dynamics of species activation in the experimental data (Table S1). The Netflux software (available on GitHub at https://github.com/saucermanlab/Netflux) has been used to generate a system of LDEs.

### Statistics

All data are presented as the mean ± SEM. Analysis of experimental conditions in NRCMs considered two distinct cardiomyocyte isolations and multiple conditions, with multiple wells in each experiment. For this reason, a two-way ANOVA followed by Tukey post-hoc test for multiple comparisons was selected for statistical calculation. In hiPSC-CMs and CM^2^ independent student t-tests were performed. For all analyses, a *P*-value < 0.05 was considered statistically significant.

## Supplementary Material

Supplement 1

1

## Highlights

- Reverse-phase protein arrays capture distinct signatures of cellular signaling in response to diverse hypertrophic ligands.
- Partial least squares regression model maps proteomic signatures to diverse patterns of cell morphology and gene expression.
- Neuregulin-1 induces elongation of rodent- and human-derived cardiomyocytes and markers of physiological hypertrophy.
- PI3K mediates both neuregulin-1-induced elongation and cell area, validating the PLSR model. In contrast, p38 regulates cell area but not elongation.
- Logic-based model demonstrates that the characterized mechanisms are sufficient to predict how distinct PI3K and p38 dynamics drive size and shape.
