# Supplementary material for: Systems analysis reveals neuregulin-1 control of cardiomyocyte size and shape mediated by distinct PI3K and p38 pathways
Source: bioRxiv. 2025 Oct 3:2025.10.01.679873. Preprint. [Version 1] doi: 10.1101/2025.10.01.679873 (PMC12621799; doi:10.1101/2025.10.01.679873)
Supplement: 1 [file NIHPP2025.10.01.679873V1-supplement-1.pdf]

bioRxiv preprint doi: https://doi.org/10.1101/2025.10.01.679873; this version posted October 3, 2025. The copyright holder for this preprint (which was not certified by peer review) is the author/funder, who has granted bioRxiv a license to display the preprint in perpetuity. It is made available under aCC-BY 4.0 International license.

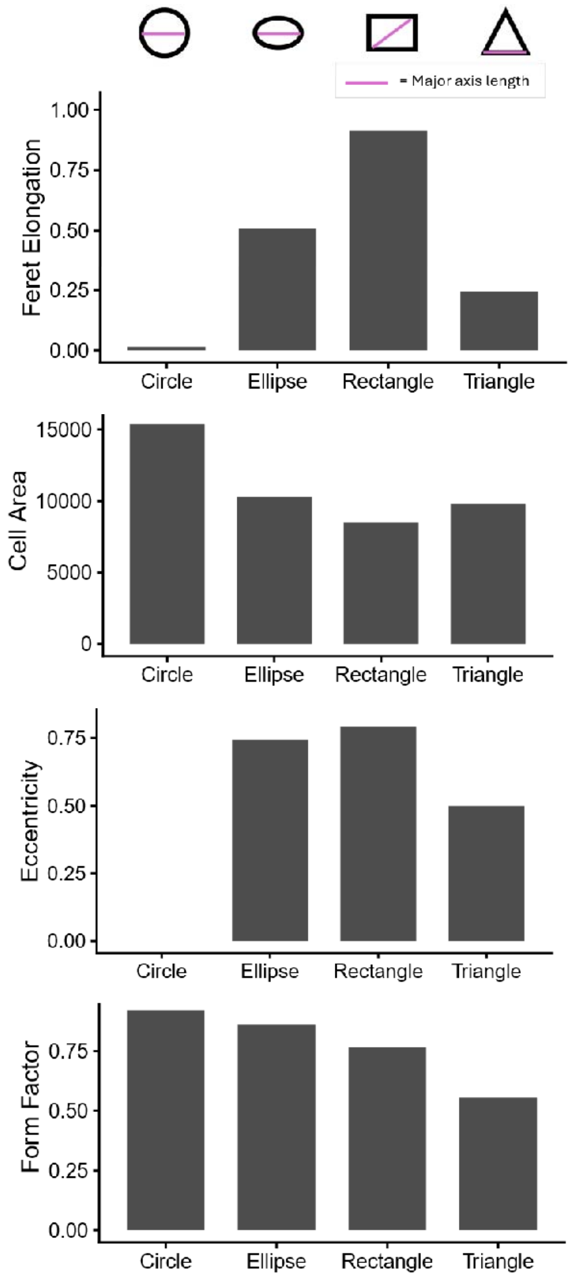

Supplemental Figure 1: Quantifications using cell morphological features in idealized shapes; circle, ellipse, rectangle, and triangle.

bioRxiv preprint doi: https://doi.org/10.1101/2025.10.01.679873; this version posted October 3, 2025. The copyright holder for this preprint (which was not certified by peer review) is the author/funder, who has granted bioRxiv a license to display the preprint in perpetuity. It is made available under aCC-BY 4.0 International license.

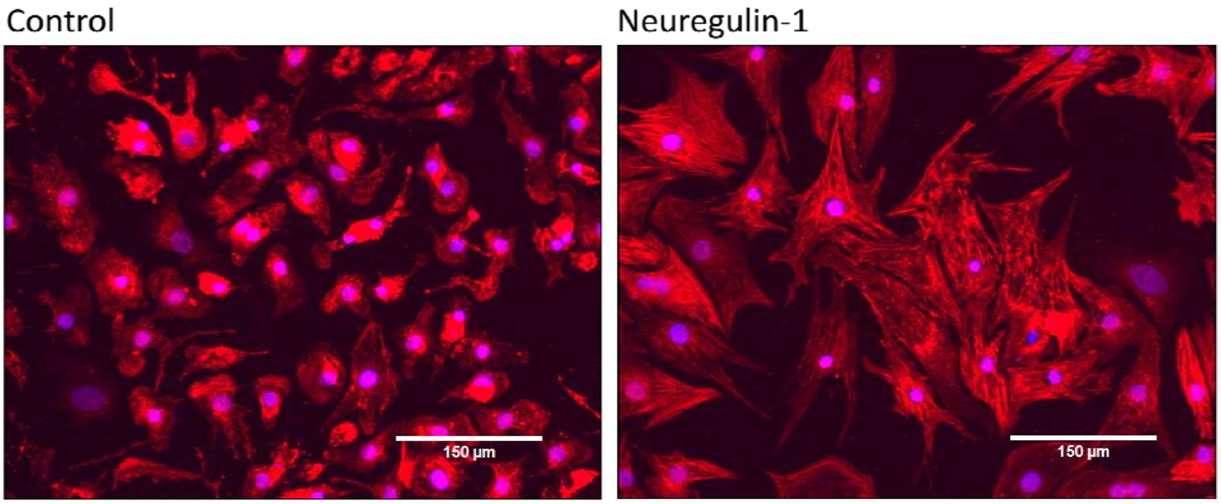

Supplemental Figure 2: Nrg1-treated neonatal rat cardiomyocytes exhibited formation of organized sarcomeres with visible myofibrils
